# Location and timing of infection drives a sex-bias in *Haemoproteus* prevalence in a hole-nesting bird

**DOI:** 10.1017/S0031182024001021

**Published:** 2024-07

**Authors:** William Jones, P. Navaneeth Krishna Menon, Anna Qvarnström

**Affiliations:** 1 Department of Animal Ecology, Evolutionary Biology Centre, Uppsala University, 75236, Sweden; 2 Department of Evolutionary Zoology and Human Biology, University of Debrecen, Debrecen, 4032, Hungary;; 3 DUW Zoology, University of Basel, Vesalgasse 1, CH-4051, Basel, Switzerland

**Keywords:** Age-pattern, disease ecology, Ficedula, Haemoproteus, Haemosporidian, parasite transmission, Plasmodium, sex-bias

## Abstract

Sex biases in prevalence of disease are often attributed to intrinsic factors, such as physiological differences while a proximate role of extrinsic factors such as behavioural or ecological differences may be more difficult to establish. We combined large-scale screening for the presence and lineage identity of avian malaria (haemosporidian) parasites, in 1234 collared flycatchers (*Ficedula albicollis*) with life-history information from each bird to establish the location and timing of infection. We found an overall infection rate of 36.2% ± 0.03 (95% CI) with 25 distinct malaria lineages. Interestingly, first-year breeding males and females had similar infection prevalence while females accrued a significantly higher infection rate than males later in life. The sex difference in infection rate was driven by the most abundant *Haemoproteus,* lineage, hPHSIB1, while the infection rate of *Plasmodium* lineages was similar in males and females. Furthermore, when infections were assigned to an apparent transmission location, we found that the sex difference in infection rate trend was driven by lineages transmitted in Europe, more specifically by one lineage (the hPHSIB1), while no similar pattern was found in African lineages. We deduce that the observed infection patterns are likely to be caused by differences in breeding behaviour, with incubating females (and nestling individuals of both sexes) being easy targets for the biting insects that are the vectors of avian malaria parasites. Overall, our results are most consistent with ecological factors rather than intrinsic factors underlying the observed sex-biased infection rate of avian malaria in collared flycatchers.

## Graphical abstract

**Figure:**
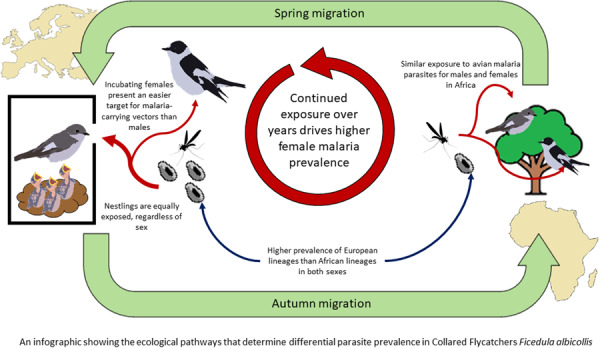

## Introduction

Sex biases in disease prevalence are increasingly recognised in studies of natural populations (Poulin, 1996; Schalk and Forbes, 1997; Moore and Wilson, 2002; Foo *et al*., 2017). Much of the scientific work on natural populations has been focused on proximate physiological reasons for differences in infection rates and susceptibility between the sexes, such as the interaction between sex hormones and the immune system (Folstad and Karter, 1992; Poulin, 1996; Zuk and McKean, 1996; Klein, 2004). Many of these intrinsic factors have been shown to skew infection biases towards males, mostly due to an antagonistic relationship between testosterone and immune function (Foo *et al*., 2017).

However, ecological factors may have a direct proximate role in causing sex-biased infection rates due to behavioural differences between the sexes that, in turn, leads to differential exposure to parasites (Tinsley, 1989; Krasnov *et al*., 2005; Zuk and Stoehr, 2010; Brown and Symondson, 2014). In a multispecies comparison of sex-specific parental roles in birds, the different breeding behaviours of males and females were suggested as a potential explanation for higher parasite prevalence in females (McCurdy *et al*., 1998). For example, in species where only 1 of the 2 sexes incubate the eggs, these stationary individuals were suggested to provide easier targets for the biting insects that transmit avian malaria than individuals of the other sex (McCurdy *et al*., 1998). Other studies have argued that this may be especially true for hole-nesting species, where incubating females were suggested to increase the number of ectoparasites and pathogen vectors in the nest by acting as ‘beacons’ of vector attracting compounds, such as CO_2_ and volatile organic compounds (Caillouët *et al*., 2013; Lutz *et al*., 2015; Castaño-Vázquez *et al*., 2020). By contrast, in open-cup nesting species male biased infection rates are more common (van Oers *et al*., 2010; Lutz *et al*., 2015; Calero-Riestra and García, 2016). However, direct links between sex-biased behaviour and the risk of infection are rarely revealed because the timing of infection often remains unknown in studies of natural populations. Here we circumvent this problem by using detailed life-history information, from a long-term study of individually marked collared flycatchers (*Ficedula albicollis*); to establish the timing of infection and to test expectations consistent with either physiological or behavioural differences being the main cause of a sex-biased infection rate.

Collared flycatchers are migratory, sexually dimorphic, passerine birds that breed across Europe and overwinter in sub-Saharan Africa (Cramp *et al*., 1993). Males and females have different behaviours at the nest, with females taking sole responsibility for incubation, while males find the nesting location and occasionally visit the nest to supplement the feeding of incubating females (Lifjeld and Slagsvold, 1986). Both sexes contribute to feeding nestlings (Pärt *et al*., 1992).

Avian malaria is a disease commonly caused by two, well studied haemosporidian parasite genera: *Haemoproteus* and *Plasmodium*. Haemosporidians (herein avian malaria) are blood-borne parasites that require both a vertebrate and an insect host to complete their life cycle and its transmission occurs globally (Valkiūnas, 2005). *Haemoproteus* is largely transmitted by *Culicoides* biting midges, many species of which directly target and prefer to feed in nest holes (Votýpka *et al*., 2009; Žiegytė *et al*., 2021), while *Plasmodium* is more frequent in mosquitoes (Valkiūnas, 2005), such as members of the genus *Culex*, which prefer to seek for hosts in the open (Ryan *et al*., 2017). Some avian malaria lineages are specific to just one host species, while others have been found in a wide range of possible hosts (Bensch *et al*., 2009; Clark *et al*., 2014; Ellis *et al*., 2020). This potential for high specificity, coupled with increasingly rich sampling efforts around the world provides the opportunity to ascertain the potential transmission zones for many avian malaria lineages. To date, at least 4500 unique lineages have been detected in 2100 avian host species from all continents, but Antarctica (Bensch *et al*., 2009; Ellis *et al*., 2019). Collared flycatchers are commonly infected with avian malaria parasites, with prevalence being as high as 40% in some populations (Kulma *et al*., 2013; Szöllősi *et al*., 2016; Jones *et al*., 2018). Additionally, an apparent transmission location has been identified for many of these lineages, with collared flycatchers gaining infections in both their breeding and non-breeding ranges (Jones *et al*., 2018). However, few studies to date, have been comprehensive enough to simultaneously investigate the patterns of avian malaria prevalence and diversity across host sex and host age categories to establish when in life and where (i.e. at the breeding grounds or at the wintering grounds) infection occur, nor have consistent patterns of sex-specific infection been detected.

In this study, we used a long-term dataset of breeding collared flycatchers and their avian malaria parasites to test whether sex-differences in parasite infection exist, how parasite prevalence changes across age categories and whether malaria lineage communities differ between the sexes. If hole-nesting behaviour imposes an increased risk of infection, we expect to find female biased infection rates solely among malaria lineages transmitted at the breeding sites in Europe and that this bias builds up across age-classes following repeated breeding events.

## Materials and methods

### Sampling and screening

Since 2002, over 2000 nest boxes have been systematically monitored for breeding collared flycatchers on the Swedish island of Öland (56°44′N 16°40′E) (Qvarnström *et al*., 2010). Between 2002 and 2016, during each breeding season (May–June), male and female flycatchers were caught at the nest and, if necessary, ring-marked and roughly 30ɥl of blood was collected from each bird and stored in ethanol. Females were mostly caught in the middle of the incubation period and males were caught approximately 10 days later while feeding nestlings. There is some evidence that prevalence can show an apparent decrease during the breeding season, even within the space of a few weeks, in collared flycatchers (Szöllősi *et al*., 2016). Therefore, sampling day (May 1^st^ = 1 – July 4^th^ = 65) was noted and included in analyses to account for any potential sampling-date bias. Smaller numbers of both sexes were caught earlier in the breeding season during the courtship period. Unringed flycatchers were aged as either 1 year old or older based on plumage features. Males, by brown, rather than black wing feathers (Svensson, 1992) and females by the shape and wear of their primary coverts (worn and pointed in first-year females, fresh and rounded in older females) (Pärt *et al*., 1992; Evans *et al*., 2011). For the purposes of this study, individuals were only screened once during their lifetime. In total, 728 individuals were included from previous studies in this system (Kulma *et al*., 2013, 2014; Jones *et al*., 2018) and 506 were newly sampled. Nestlings were not screened for avian malaria parasites, as infections are not typically detectable for several weeks after initiated (Cosgrove *et al*., 2006). However a previous study on avian malaria prevalence in fledged collared flycatchers detected some infections, suggesting that transmission is indeed occurring before they migrate to Africa (Fletcher *et al*., 2019). For a detailed list of sample sizes for each sex and age category, see Table 1. Ethical permissions were provided by the Linköping Animal Ethics Board (5-2-18—7556/14).

**Table 1.** Distribution of avian malaria lineages in collared flycatchers from Öland, Sweden with assigned transmission locations

Lineage	Genus	Transmission	Young females	Old females	Young males	Old males
COLL2	*Haemoproteus*	Unknown	6	21	1	8
COLL3	*Haemoproteus*	Europe	3	6	3	9
PFC1	*Haemoproteus*	Europe	3	7	1	2
PHSIB1	*Haemoproteus*	Europe	36	105	30	63
WW2	*Haemoproteus*	Europe	0	1	0	0
ACCTAC01	*Plasmodium*	Africa	0	2	0	0
AEMO01	*Plasmodium*	Africa	0	0	1	0
COLL10	*Plasmodium*	Unknown	0	1	0	2
COLL11	*Plasmodium*	Africa	0	2	0	0
COLL4	*Plasmodium*	Africa	2	0	0	1
COLL6	*Plasmodium*	Unknown	0	0	1	0
COLL7	*Plasmodium*	Africa	2	2	0	1
GRW07	*Plasmodium*	Unknown	0	1	0	0
GRW09	*Plasmodium*	Africa	0	1	1	1
GRW10	*Plasmodium*	Africa	0	0	0	1
GRW11	*Plasmodium*	Europe	0	1	0	0
LAMPUR03	*Plasmodium*	Africa	0	2	0	0
PBPIP1	*Plasmodium*	Unknown	1	0	0	0
RTSR1	*Plasmodium*	Africa	0	1	0	5
SGS1	*Plasmodium*	Unknown	0	1	0	3
SYBOR05	*Plasmodium*	Africa	0	0	0	1
SYBOR10	*Plasmodium*	Africa	0	2	0	1
TERUF02	*Plasmodium*	Africa	1	0	0	1
TURDUS1	*Plasmodium*	Europe	0	1	0	0
WW4	*Plasmodium*	Africa	1	2	0	0
Unidentified	NA	Unknown	13	28	18	41

DNA was extracted *via* the high salt technique (Aljanabi and Martinez, 1997). Briefly, blood was digested overnight in a solution of proteinase K, SDS and Tris and EDTA buffers. DNA was precipitated out using 6 M NaCl and 99% ethanol. Extracted DNA was stored in TE buffer. DNA concentration was quantified using a NanoDrop2000 (Thermo Scientific) and diluted to a concentration of approximately 25 ng *μ*L^−1^. To ascertain infection status, DNA extracts were screened for *Plasmodium* and *Haemoproteus* presence using an established nested PCR technique, targeting a fragment of the cytochrome b mitochondrial gene, using 2 sets of primer pairs (Waldenström *et al*., 2004). Firstly, primers HAEMNF and HAEMNR_2_, which amplify a 580 base pair DNA fragment, followed by a second round with the primers HAEMF and HAEMR_2_, which amplify a final 478 base pair fragment. Negative (ddH2O) and positive controls were included to control for possible contamination and amplification failure during PCRs. PCR products were then stained with GelGreen and visually inspected for malaria presence or absence on a 1.5% agarose gel. Positive samples were Sanger sequenced to ascertain lineage identity. Sequences were aligned using Mega7 software and compared with previously published sequences in the publicly available MalAvi database, which collates lineage-specific infection records from around the world (Bensch *et al*., 2009). Confirmed multiple infections in a single host were rare (2/1234). Both of these individuals were infected with 2 *Haemoproteus* lineages (hCOLL3 and hPHSIB1), as both of these lineages are also transmitted in Europe, they were treated as a single infection in our analyses. Furthermore, Individuals for which lineage could not be ascertained (100/1234) could, in some cases, refer to sequencing failure due to multiple infections.

### Assigning transmission location

Records of infection from all lineages detected in the individuals from this study were extracted from the MalAvi database. In total, 293 host species were found to share malaria lineages with collared flycatchers. Infection information from captive birds or from experimental infections were removed from the analysis, as were records flagged as likely contamination errors (Bensch *et al*., 2021). The ranges for each host species were categorised by ecozone, as defined by Schultz (1995), using range maps from the ‘Birds of the World’ website (Billerman *et al*., 2021). Each species was determined to be either migratory (individuals moved between ecozones) or resident (any movements remained within 1 ecozone). Lineages with likely transmission on the breeding grounds were determined if the lineage had been detected in resident species in the Palearctic ecozone. Lineages that were not detected in resident, Palearctic host species were determined to be transmitted during the non-breeding season (Jones *et al*., 2018). A full table of lineages and their apparent transmission zones can be found in the supplementary materials (Table S2).

Individuals from which sequences were unable to be resolved (100 individuals) were included in analyses of overall infection prevalence but excluded from transmission specific analyses. A complete list of infection records can be found in Table 1.

### Statistical analyses

All analyses were conducted using R version 4.0.5 (R Development Core Team, 2021). To investigate the patterns of malaria prevalence in collared flycatchers, we constructed 8 generalised linear mixed-effects models, exploring: a, overall infection prevalence; b, infection prevalence of European-transmitted lineages; c, infection prevalence of African-transmitted lineages; d, infection prevalence of lineages with unknown-transmission; e, infection prevalence of all *Haemoproteus* lineages; f, infection prevalence of hPHSIB1 (the most abundant lineage); g, infection prevalence of all other *Haemoproteus* lineages and h, infection prevalence of *Plasmodium* lineages using the package ‘lme4’ (Bates *et al*., 2015) with binomial error structures and a logit links. Models had the same error structure with binomial fixed effects of sex and age (young = 1^st^ breeding year and old ⩾2^nd^ breeding year) and the interaction between the 2. Furthermore, sampling day was included as a fixed effect, as models including it as a random effect returned a singular fit. Finally, year was included as a random variable. Models exploring African-transmitted and *Plasmodium* lineages failed to converge, therefore generalised linear models, without random effects, were employed instead. Estimates of effect sizes (odds ratios) were calculated for infection probabilities between age and sex classes to confirm the strength of association between infection and age and infection and sex.

To compare lineage diversity between the sexes, we calculated the Shannon diversity indices and compared them using a Hutcheson *t*-test, using the package ‘vegan’ (Hutcheson, 1970; Dixon, 2003). Hutcheson *t*-tests are a modified version of the classic *t*-test that provides a method to compare 2 samples by incorporating the variance of the Shannon diversity index measures. To test whether lineage communities in young or old and male or female flycatchers was structured or not, we used analyses of similarity (ANOSIMs) in package ‘vegan’. ANOSIM detects differences between 2 or more sampling units by comparing between group dissimilarity with the mean of within group dissimilarity. All tests were done using a ‘Bray–Curtis’ dissimilarity matrix with 99 999 iterations. As well as a *P* value, ANOSIM provides an *R* statistic that indicates the extent to which groups are separated. *R* values near to 0 suggest community similarity while *R* values close to 1 suggest complete community dissimilarity. In addition, indicator lineages, i.e. lineages that occur more frequently in 1 group than expected were detected using the ‘mulitplatt’ function in the package ‘indicspecies’ (De Cáceres *et al*., 2016). Finally, to visualise the relatedness of the different lineages and their relative proportions in each sex, a minimum-spanning network (Bandelt *et al*., 1999) of detected lineages was created by using the 478 base-pair cytochrome b DNA fragment in the program PopART (Leigh and Bryant, 2015).

## Results

**Figure 1.**
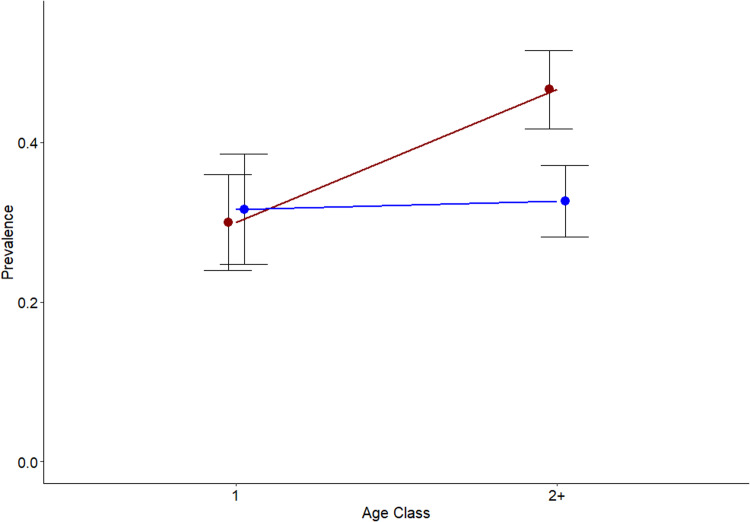
Overall avian malaria prevalence across age classes in male (blue) and female (red) collared flycatchers with 95% confidence intervals. First year individuals experience similar infection rates, however older females experience a higher risk of infection.

From 1234 (628 female, 606 male) adult collared flycatchers screened for infections with *Plasmodium* and *Haemoproteus* we found an overall parasite prevalence of 36.6% by 25 distinct lineages, amounting to 20 *Plasmodium* and 5 *Haemoproteus* lineages. Infection rate in first year individuals was not significantly different between males and females but older females had a significant rise in malaria prevalence. This was not observed among male age classes (Fig. 1, Table 2a). Odds ratio calculations suggested that older females were 30% more likely to be infected than first year females (odds ratio: 2.041, 95% CI: 1.428–2.935, *p* < 0.001).

**Table 2.** Generalised linear mixed-effects models (GLMM) and generalised linear effects models (GLM) evaluating the role of age and sex in explaining malaria infection prevalence including; (a) all infections (b) only European-transmitted infections (c) only African-transmitted infections (d) only infections with unknown-transmission location (e) only *Haemoproteus* infections (f) only hPHSIB1 infections (g) only non-hPHSIB1 *Haemoproteus* infections, and (h) only *Plasmodium* infections in collared flycatchers. Significant values highlighted thusly (*>0.05; **>0.01; ***>0.001)

	Random effects (year)		Fixed effects				
Model	Variance	Standard deviation	Fixed effect	Estimate	Standard error	z-value	*P* value
(a) All infections	0.366	0.605	Intercept	−0.565	0.483	−1.169	0.243
			age	0.738	0.188	3.932	0.001***
			sex	−0.173	0.633	−0.274	0.785
			sampling date	−0.013	0.013	−1.007	0.314
			age*sex	−0.549	0.275	−1.997	**0**.**046**
			sampling date * sex	0.007	0.016	0.466	0.641
(b) European infections	0.510	0.714	Intercept	−1.574	0.417	−3.777	<0.001***
			age	0.729	0.217	3.365	0.001***
			sex	−0.004	0.288	−0.015	0.988
			sampling date	−0.003	0.010	−0.279	0.780
			age*sex	−0.643	0.322	−1.995	0.046*
(c) African infections	-	-	Intercept	−3.465	0.775	−4.468	<0.001***
			age	0.304	0.498	0.610	0.542
			sex	−0.782	0.855	−0.914	0.361
			sampling date	−0.002	0.020	−0.124	0.902
			age*sex	0.610	0.917	0.665	0.506
(d) Unknown transmission	0.344	0.587	Intercept	−1.830	0.451	−4.057	<0.001***
			age	0.356	0.285	1.252	0.210
			sex	0.348	0.360	0.965	0.335
			sampling date	−0.006	0.011	−0.593	0.553
			age*sex	−0.307	0.401	−0.765	0.444
(e) *Haemoproteus* infections	0.658	0.811	Intercept	−1.102	0.415	−2.658	0.008**
			age	0.751	0.207	3.621	<0.001***
			sex	−0.030	0.279	−0.106	0.915
			sampling date	−0.016	0.009	−1.680	0.093
			age*sex	−0.628	0.312	−2.014	0.044*
(f) hPHSIB1 infection	0.647	0.805	Intercept	−1.830	0.451	−4.057	<0.001***
			age	0.702	0.226	3.101	0.002**
			sex	0.012	0.301	0.041	0.967
			sampling date	−0.005	0.010	−0.446	0.655
			age*sex	−0.671	0.336	−1.999	0.046**
(g) non-hPHSIB1 *Haemoproteus* infections	0.096	0.310	Intercept	−2.009	0.548	−3.669	<0.001***
			age	0.432	0.351	1.233	0.218
			sex	−0.333	0.562	−0.593	0.553
			sampling date	−0.029	0.014	−2.038	0.042*
			age*sex	−0.084	0.619	−0.136	0.892
(h) *Plasmodium* infections	-	-	Intercept	−3.509	0.701	−5.004	<0.001***
			age	0.451	0.452	0.998	0.318
			sex	−0.634	0.728	−0.871	0.384
			sampling date	0.002	0.018	0.104	0.917
			age*sex	0.430	0.777	0.553	0.580

**Figure 2.**
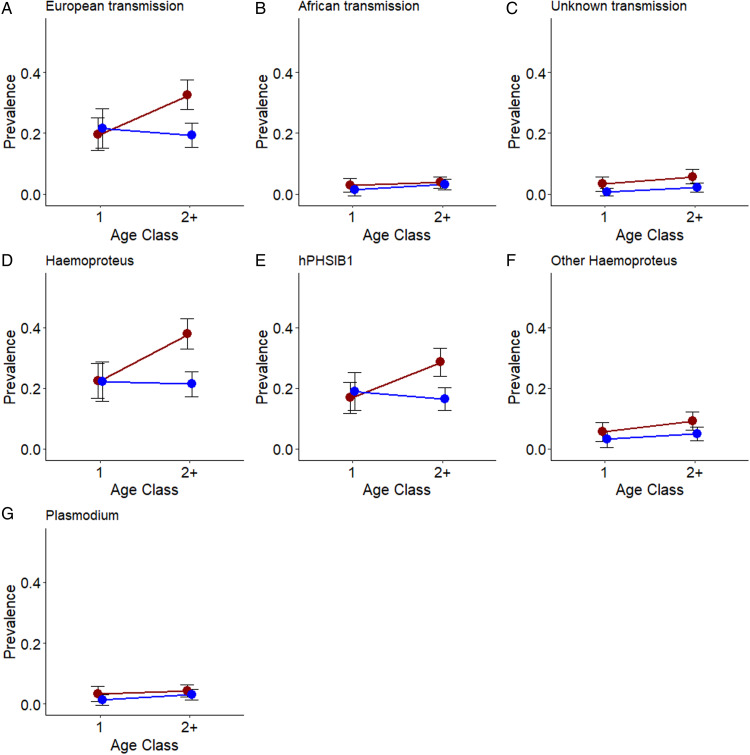
Prevalence of European-transmitted (A), African-transmitted (B), lineages of unknown transmission (C), *Haemoproteus* (D), hPHSIB1 (E), non hPHSIB1 *Haemoproteus* infections, and *Plasmodium* (G) lineages in male (blue) and female (red) collared flycatchers with 95% confidence intervals. Older females experience a significant increase in infection risk with European, overall *Haemoproteus* and hPHSIB1 lineages. Both sexes experience a similar rate of increase in African, unknown-transmission, non hPHSIB1 and *Plasmodium* lineages over time.

When infections were classified by apparent transmission location, we found that, the female biased age-dependent rise in malaria prevalence was driven by infections with European lineages (Fig. 2A, Table 2b). Both sexes were more likely to be infected with African lineages with increasing age but there was no significant effect of sex or the interaction between age and sex on the likelihood of infection with African lineages (Fig. 2B, Table 2c), and the same was true for *Plasmodium* infections (Table 2d). Our analyses of infection prevalence of all *Haemoproteus* lineages and of hPHSIB1 infections specifically both revealed the same difference between the sexes with females being significantly more likely to be infected due to an increased infection rate with age that was absent in males. There were no significant effects of age or sex in the model investigating infection patterns of *Haemoproteus* lineages when infection with hPHSIB1 were excluded, implying that it is the most common lineage, i.e. hPHSIB1, that is the main driver of the overall female biased infection rate. There were also no significant differences in *Plasmodium* infection prevalence between the 2 sexes (Table 2e–h). Sampling day did not have any significant effect on infection prevalence in any of the models.

**Figure 3.**
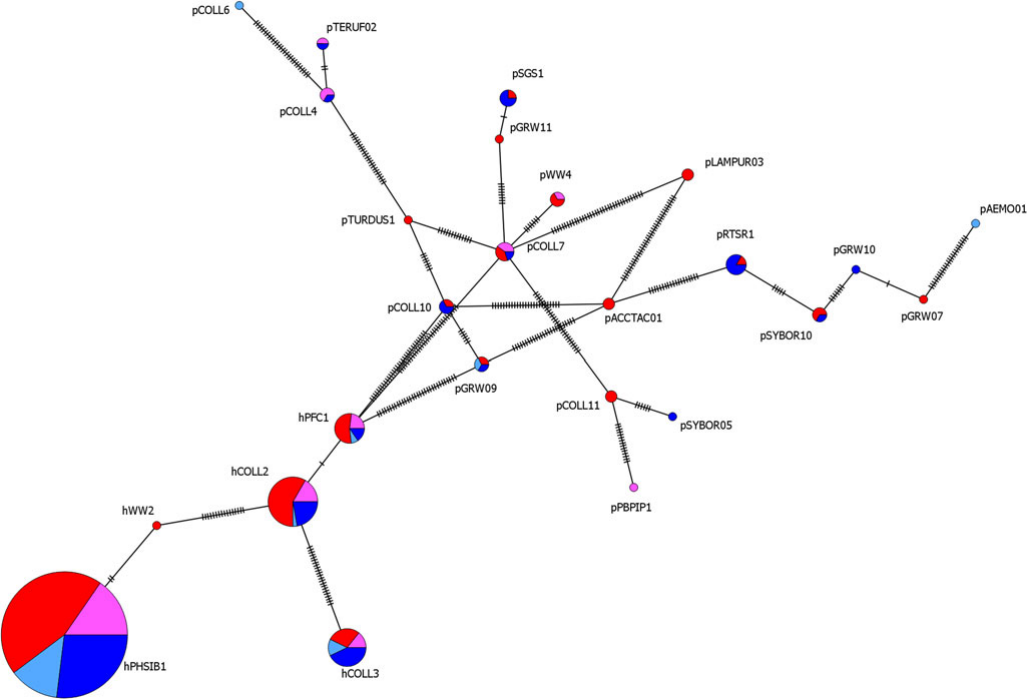
Minimum-spanning network of mistochondrial haplotypes of avian malaria lineages, based on a 478 base pair cytochrome b fragment. The size of each haplotype represents the number of individual collared flycatchers carrying that particular lineage. The colours denote the sex and age of the individuals: first-year male (light blue), adult male (dark blue) and young fesmale (pink) and adult female (red).

We found fewer overall lineages in males than in females (16 *vs* 21), however this did not translate to a significant difference in the Shannon diversity of malarial lineages between males (± 95% CI) (*H* = 1.354 ± 0.259) or females (*H* = 1.416 ± 0.215), (Hutcheson *t*-test: *t* *=* *0.367; df* *=* *300; p* *=* *0.714*). Lineage diversity tended to be higher in older flycatchers (22 lineages; *H* = 1.475 ± 0.199) than younger birds (12 lineages; *H* = 1.208 ± 0.297), however this was not statistically significant (Hutcheson *t*-test: *t* *=* *1.497; df* *=* *181; p* *=* *0.136*). Finally, malarial lineage communities were not structured in collared flycatchers either by age (*R* *=* *−0.5, p* *=* *1.000*) or sex (*R* *=* *1, p* *=* *0.333*), with all of the most common lineages were shared between age and sex groups (Fig. 3, Table S3).

## Discussion

Few previous studies have assessed age-dependant malaria prevalence. Two such examples are a study of common house martins (*Delichon urbicum*) showing a general increase (Marzal *et al*., 2016) and a study on Seychelles warblers showing a general decline (van Oers *et al*., 2010; Hammers *et al*., 2016) in parasite prevalence with age across both sexes. Parasite prevalence is often expected to decrease across age groups, as the most susceptible individuals die young and the surviving older individuals develop immunity to the parasite (van Oers *et al*., 2010; De Nys *et al*., 2013; Lynsdale *et al*., 2017). By contrast, temporarily or even consistent increases, as observed in the study on house martins, can be expected when parasite taxa that are more benign predominate, and when infections are chronic leading to a build-up of infections as the total time of possible exposure increases (Wood *et al*., 2013). In our study, we find that malaria infection rates increase with age, but only in females and only when *Haemoproteus* infections acquired in Europe are considered. Importantly, we find no sex-difference in infection rate among the first-year breeders. Our analyses furthermore reveal that it is the most abundant *Haemoproteus* lineage, hPHSIB1, that drives the observed sex-different infection pattern, with females being significantly more likely to be infected later in life than males (Table 2f). The crucial question then becomes whether the observed female-biased built up of malaria prevalence proximately is caused by physiological or behavioural differences between the 2 sexes. While untested sex-specific physiological factors may have some influence on these patterns (Hasselquist *et al*., 2007), we argue that several lines of evidence allow us to reject the proximate physiological explanation as the main driver of sex-specific haemosporidian infection patterns in collared flycatchers. There is a time-lag between the event when the bird gets the infection due to a vector bite and the advanced stage of infection needed for detection of an ongoing infection based on the methods that we have used (Valkiūnas, 2005; Cosgrove *et al*., 2006). This means that the vast majority of first year breeding birds with verified positive infection status with European lineages were infected during the preceding short time-window between hatching and migrating to the non-breeding grounds (Fletcher *et al*., 2019). Insect vectors, particularly biting midges from the genus *Culicoides* are known to target nests and nesting birds, particularly those of hole-nesting species (Martínez-De La Puente *et al*., 2009; Votýpka *et al*., 2009; Caillouët *et al*., 2012; Žiegytė *et al*., 2021). As a result, male and female nestlings and, to a lesser extent, fledglings likely present a similar target to biting insects since there are no major sex differences in host-behaviour during this period (Cozzarolo *et al*., 2019). The lack of a difference in infection rate between first year males and females therefore suggests that any intrinsic physiological differences in attracting infections between male and female collared flycatcher nestlings are small or even slightly male biased (as we find a slight non-significant male biased infection among first-year breeding birds) (Burkett-Cadena *et al*., 2010).

Sex differences in composition of malaria lineages associated with corresponding variation in virulence may result in higher observed prevalence in the sex carrying the most benign lineages as selective removal would occur in the sex carrying the most virulent lineages. However, we find no strong evidence for differing avian malaria communities in the 2 sexes, with lineage communities in males *vs* females being entirely unstructured (Fig. 3), with all the most abundant lineages being shared between the sexes. In addition, while 1 study found no sex-specific survival difference for infected collared flycatchers (Kulma *et al*., 2014), more recent work has found that female collared flycatchers, and not males, may actually suffer a subtle increase in mortality when infected with haemosporidians (Jones, 2019). This means that we can rule out selective removal of infected males as a possible explanation to the observed female bias in the built up of prevalence across age classes. We moreover consider sex-biased clearance to be an unlikely explanation to these results. Relatively short-lived bird species such as collared flycatchers and common house martins are not expected to invest as many resources into managing and clearing infections as longer-lived species such as the Seychelles warbler where clearance has been observed (Miller *et al*., 2007). Anecdotally, we have observed rare incidences of apparent infection clearances in both sexes (females 8%, males 6% of repeated birds), although whether this is due to poor detection of dormant infections or whether birds really clear the infections remains to be investigated (Jarvi *et al*., 2002). This fact thereby rules out more effective clearance of disease by males as a likely explanation to the sex-difference in age-dependent prevalence that we find.

Sex-differences in breeding ecology and associated behaviours may be a major cause of differences in exposure to vectors, which in turn may result in higher frequency of infections observed among females following breeding events. Females spend more time inside the nest, incubating the eggs and keeping newly hatched nestling warm, meaning that they may be more exposed to vectors than males (Martínez-De La Puente *et al*., 2009; Tomás *et al*., 2020). Studies on other species have found that females have elevated metabolic rates during incubation and as an increased metabolic rate increases the quantity of vector attractive compounds such as CO_2_ and volatile organic compounds, this makes females more of a target (De Heij *et al*., 2007; Nord *et al*., 2010).

We find that male collared flycatchers mainly obtain avian malaria early in life, most likely during their stationary nestling stage while females maintain a high risk of infection throughout their lives following repeated breeding events. Taken together, these findings are most compatible with a difference in the breeding behaviours of males and females playing a major role in explaining the observed overall sex-biased infection rate. This conclusion is further supported by the fact that we only find a female specific age-dependent built up of infections of malaria lineages transmitted in Europe while a similar pattern is not found when considering malaria lineages transmitted in Africa. We also argue that a bias in sampling time of males and females is an unlikely alternative explanation to the observed sex difference in age-dependent infection patterns. This is because we find very little evidence for a change in infection patterns over the course of single breeding season in almost all cases (with the exception of a small, but significant decrease in some of the rarer *Haemoproteus* infections over time), a pattern also detected in another collared flycatcher population (Szöllősi *et al*., 2016).

Further work still needs to be done to resolve the transmission zones of several of the malarial lineages detected in this study. For some lineages, such as pSGS1, transmission appears to occur globally (Bensch *et al*., 2009; Marzal *et al*., 2015) making it impossible to infer where flycatchers were infected with this lineage. It is possible that some of the lineages currently determined to be transmitted in Africa may also be transmitted in Europe or vice-versa. Furthermore, while fewer studies have collected samples in Africa than in Europe, there have still been several important community-wide studies that have given a clear picture of lineage prevalence and range, at least for the most common lineages (Loiseau *et al*., 2012; Lutz *et al*., 2015; Tchoumbou *et al*., 2020). Yet, further work is still needed to ascertain the exact parameters for successful transmission for these most abundant lineages. However, we think it is unlikely that such changes in transmission classification will result in changes to our interferences on sex-biases in infection risk in collared flycatchers, due to the scarcity of many of the lineages. Nevertheless, there remains a large gap in our knowledge regarding the exact transmission pathways of hPHSIB1, the most abundant lineage in collared flycatchers. This lineage appears to be most common at higher latitudes in the Palearctic (Huang *et al*., 2018; Jones *et al*., 2018), suggesting dependence on more northerly vectors or colder climates for successful transmission, thereby making it unlikely that transmission also occurs in Africa. A recent study found hPHSIB1 sporozoites in the salivary glands of the biting midge *Culicoides segnis* (Chagas *et al*., 2022). However, *C. segnis* is a relatively uncommon species in Scandinavia (Ander *et al*., 2012), and given the abundance of hPHSIB1 in collared flycatchers in our population and its presence in several other Eurasian passerines (Hellgren *et al*., 2007; Palinauskas *et al*., 2013; Ellis *et al*., 2020), it is likely to exploit several other vector species too.

Our results show that there are considerable underlying differences in parasite infection rates across sex and age categories, with female collared flycatchers having an overall greater risk of contracting avian malaria. This sex-difference is almost entirely driven by the most abundant *Haemoproteus* lineage, hPHSIB1 that is transmitted in Europe. Higher parasite prevalence in females appears to be an unusual trend in birds and highlights how idiosyncrasies in ecology or behaviour may produce contrary patterns. Future studies need to focus on the role of malaria vectors in the transmission process, to fully understand the dynamics and impacts of vector transmitted diseases. We conclude that our results are most compatible with the observed sex-differences in parasite prevalence in collared flycatchers being driven by differences in reproductive behaviours that, in turn, leads to higher female exposure to vectors. Behaviourally driven differences in exposure to vectors are often overlooked but can have strong implications for immunological, conservation or ecological research.

## Supporting information

Jones et al. supplementary material Jones et al. supplementary material

## Data Availability

Data is available on request from the authors.

## Supplementary material

The supplementary material for this article can be found at https://doi.org/10.1017/S0031182024001021.
