# Supplementary material for: Location and timing of infection drives a sex-bias in Haemoproteus prevalence in a hole-nesting bird
Source: Parasitology. 2024 Oct 21;151(8):875–83. doi: 10.1017/S0031182024001021 (PMC11578888; doi:10.1017/S0031182024001021)
Supplement: Jones et al. supplementary material [file S0031182024001021sup001.docx]

**Supplementary materials**

**Location and timing of infection drives a sex-bias in *Haemoproteus* prevalence in a hole-nesting bird**

Table S1 Sample sizes across years of collared flycatchers in this study.

| Year | 1 Females | 2+ Females | 1 Males | 2+ Males | Total |
| --- | --- | --- | --- | --- | --- |
| 2002 | 0 | 12 | 4 | 11 | 27 |
| 2003 | 16 | 17 | 18 | 12 | 63 |
| 2004 | 56 | 47 | 34 | 48 | 185 |
| 2005 | 32 | 39 | 31 | 36 | 138 |
| 2006 | 6 | 5 | 4 | 5 | 20 |
| 2007 | 13 | 21 | 12 | 20 | 66 |
| 2009 | 25 | 72 | 22 | 70 | 189 |
| 2010 | 20 | 30 | 19 | 32 | 101 |
| 2011 | 8 | 8 | 6 | 7 | 29 |
| 2012 | 19 | 41 | 3 | 39 | 102 |
| 2013 | 11 | 22 | 10 | 17 | 60 |
| 2014 | 5 | 12 | 2 | 17 | 36 |
| 2015 | 0 | 2 | 1 | 9 | 12 |
| 2016 | 16 | 73 | 11 | 106 | 206 |
| Total | 227 | 401 | 177 | 429 | 1234 |

Table S2. Determination of the transmission location for each of the 25 lineages detected in collared flycatchers. The number of migratory or resident hosts for each ecozone for each lineage are indicated. *COLL2 was detected in a hatch year song thrush (*Turdus philomelos*) in Sweden ^1^, indicating transmission in Europe as well as in Africa, furthermore song thrushes rarely overwinter outside Europe. **COLL3 was only detected in migratory species, however as the lineage was detected in fledglings before their first visit to Africa, this lineage is determined to be transmitted on the breeding grounds ^2^. ***GRW11 was detected in cape bulbuls (*Pycnonotus capensis*) in South Africa ^3^, however this is likely the result of a biological invasion, given that the lineage is widespread in several resident species in Europe that have been successfully introduced to southern South Africa. Given that the range of the cape bulbul does not overlap with the wintering range of collared flycatchers and given that the environmental conditions in southern South Africa resemble southern Europe, it is likely that transmission in tropical Africa is unlikely for this lineage. Additional details are available from the publicly accessible MalAvi Database (http://130.235.244.92/Malavi/).

| **Lineage** | **Hosts** | **Status** | **Ecozone** | **References** |
| --- | --- | --- | --- | --- |
| ACCTAC01 | 17 | Resident | Afrotropical | ^3–6^ |
| ACCTAC01 | 4 | Migrant | Palearctic | ^2,7–13^ |
| AEMO01 | 1 | Migrant | Afrotropical | ^14^ |
| AEMO01 | 16 | Resident | Afrotropical | ^5,6,15–17^ |
| AEMO01 | 5 | Migrant | Palearctic | ^2,7,9,13,18^ |
| COLL10 | 1 | Migrant | Palearctic | ^9–11^ |
| COLL11 | 4 | Resident | Afrotropical | ^6^ |
| COLL11 | 1 | Migrant | Palearctic | ^9–11^ |
| COLL2 | 1 | Migrant | Afrotropical | ^6^ |
| COLL2 | 3 | Resident | Afrotropical | ^4,6,16,19^ |
| COLL2 | 1 | Resident | Australasia | ^20^ |
| COLL2 | 6 | Migrant | Nearctic | ^21–23^ |
| COLL2 | 7 | Resident | Oriental | ^24–27^ |
| COLL2 | 5 | Migrant | Palearctic* | ^1,2,9–13,16,18,28–31^ |
| COLL3 | 5 | Migrant | Palearctic** | ^1,2,9–13,16,18,28–30,32^ |
| COLL4 | 3 | Resident | Afrotropical | ^15,33^ |
| COLL4 | 2 | Migrant | Nearctic | ^23,34^ |
| COLL4 | 8 | Resident | Neotropical | ^35–37^ |
| COLL4 | 2 | Migrant | Palearctic | ^9–11,18,28,38^ |
| COLL6 | 3 | Migrant | Palearctic | ^2,9,11,13,39,40^ |
| COLL7 | 18 | Resident | Afrotropical | ^3,5,6,15,17,33,41–43^ |
| COLL7 | 2 | Migrant | Palearctic | ^2,9–12,18,28^ |
| GRW07 | 2 | Migrant | Palearctic | ^9–11,44^ |
| GRW09 | 72 | Resident | Afrotropical | ^3–6,15–17,33,41,42,45–48^ |
| GRW09 | 1 | Resident | Oriental | ^41^ |
| GRW09 | 13 | Migrant | Palearctic | ^1,2,7,9–13,16,18,28–30,39,44,49–53^ |
| GRW10 | 4 | Resident | Afrotropical | ^6,15^ |
| GRW10 | 4 | Migrant | Palearctic | ^9,11,12,28,44,54,55^ |
| GRW11 | 1 | Migrant | Afrotropical | ^14,16^ |
| GRW11 | 1 | Resident | Afrotropical*** | ^3^ |
| GRW11 | 20 | Migrant | Palearctic | ^2,9,10,12,16,18,29,32,39,49,53,55–67^ |
| GRW11 | 22 | Resident | Palearctic | ^1,3,29,32,42,49,50,54,55,57,67–94^ |
| LAMPUR03 | 19 | Resident | Afrotropical | ^4,15^ |
| LAMPUR03 | 4 | Migrant | Palearctic | ^2,9–11,30,51,95^ |
| PBPIP1 | 4 | Resident | Afrotropical | ^6,16,43^ |
| PBPIP1 | 1 | Resident | Oriental | ^24^ |
| PBPIP1 | 3 | Migrant | Palearctic | ^1,2,10,96^ |
| PBPIP1 | 1 | Resident | Palearctic | ^89^ |
| PFC1 | 2 | Migrant | Palearctic | ^1,2,9–12,16,18,28,29,88,97–99^ |
| PFC1 | 1 | Resident | Palearctic | ^16^ |
| PHSIB1 | 1 | Migrant | Afrotropical | ^100^ |
| PHSIB1 | 14 | Migrant | Palearctic | ^1,2,9–12,16,29,44,98,101,102^ |
| PHSIB1 | 12 | Resident | Palearctic | ^1,16,29,71,79,88,91,92,98,101,103^ |
| RTSR1 | 1 | Migrant | Afrotropical | ^16^ |
| RTSR1 | 3 | Resident | Afrotropical | ^43,104^ |
| RTSR1 | 13 | Migrant | Palearctic | ^1,2,6,8–12,14,16,18,29,31,32,44,49,50,55,61,65,67,105–110^ |
| SGS1 | 3 | Migrant | Afrotropical | ^16,100^ |
| SGS1 | 15 | Resident | Afrotropical | ^3,15,16,81,100,111^ |
| SGS1 | 2 | Resident | Australasia | ^112,113^ |
| SGS1 | 1 | Migrant | Nearctic | ^114^ |
| SGS1 | 2 | Resident | Nearctic | ^115^ |
| SGS1 | 9 | Resident | Neotropical | ^116,117^ |
| SGS1 | 1 | Resident | Oriental | ^41^ |
| SGS1 | 31 | Migrant | Palearctic | ^1,2,9–11,13,14,16,29,31,32,44,49–51,53,54,57,59,61–67,82,94,95,98,110,118–124^ |
| SGS1 | 55 | Resident | Palearctic | ^1,7,16,29,32,41,49,50,54,55,57,64,67–71,73–94,110,119,125–140^ |
| SYBOR05 | 1 | Resident | Afrotropical | ^141^ |
| SYBOR05 | 2 | Migrant | Palearctic | ^11,14^ |
| SYBOR10 | 1 | Migrant | Afrotropical | ^14,16^ |
| SYBOR10 | 1 | Resident | Afrotropical | ^43^ |
| SYBOR10 | 4 | Migrant | Palearctic | ^8–11,55^ |
| TERUF02 | 1 | Resident | Afrotropical | ^4^ |
| TERUF02 | 3 | Migrant | Palearctic | ^9–11,18,28,60^ |
| TURDUS1 | 1 | Migrant | Afrotropical | ^14,16^ |
| TURDUS1 | 23 | Migrant | Palearctic | ^1,2,9–11,39,49,52,60,63,94,98,100,108,110,120,123,126,127,142–145^ |
| TURDUS1 | 19 | Resident | Palearctic | ^1,16,29,38,69,71,76,79,84–88,90,91,98,101,110,125,126,128,131,134,139,144,146,147^ |
| WW2 | 23 | Migrant | Palearctic | ^1,2,8,9,11,14,16,29,44,49,52,55,61,63,67,101,148–150^ |
| WW2 | 9 | Resident | Palearctic | ^1,16,29,71,79,85,91,94,101^ |
| WW4 | 7 | Resident | Afrotropical | ^6,15,33^ |
| WW4 | 5 | Migrant | Palearctic | ^1,9–11,13,18,61,67,149^ |

Table S3. Investigation into the presence of indicator lineages in collared flycatchers, determining whether malaria communities are structured across flycatcher sex and age categories using the ‘indicspecies’ package in R. We found no significant indicator lineages in collared flycatcher communities.

| **Grouping** | **Lineage** | **Indicator value** | ***p*** |
| --- | --- | --- | --- |
| 2+ Females | hCOLL2 | 0.938 | 1.000 |
| 2+ Females & Males | hCOLL3 | 0.905 | 1.000 |
| 2+ Females | hPFC1 | 0.951 | 1.000 |
| 2+ Females | hPHSIB1 | 0.907 | 1.000 |
| 2+ Females | hWW2 | 1.000 | 1.000 |
| 2+ Females | pACCTAC01 | 1.000 | 1.000 |
| 1 Males | pAEMO01 | 1.000 | 1.000 |
| 2+ Females & Males | pCOLL10 | 0.905 | 1.000 |
| 2+ Females | pCOLL11 | 1.000 | 1.000 |
| 1 Females & 2+ Males | pCOLL4 | 0.905 | 1.000 |
| 1 Males | pCOLL6 | 1.000 | 1.000 |
| Females | pCOLL7 | 0.905 | 1.000 |
| 2+ Females | pGRW07 | 1.000 | 1.000 |
| 1 Males & 2+ Females & 2+ Males | pGRW09 | 1.000 | 1.000 |
| 2+ Males | pGRW10 | 1.000 | 1.000 |
| 2+ Females | pGRW11 | 1.000 | 1.000 |
| 2+ Females | pLAMPUR03 | 1.000 | 1.000 |
| 1 Females | pPBPIP1 | 1.000 | 1.000 |
| 2+ Males | pRTSR1 | 0.980 | 1.000 |
| 2+ Males | pSGS1 | 0.943 | 1.000 |
| 2+ Males | pSYBOR05 | 1.000 | 1.000 |
| 2+ Females & Males | pSYBOR10 | 0.905 | 1.000 |
| 1 Females & 2+ Males | pTERUF02 | 1.000 | 1.000 |
| 2+ Females | pTURDUS1 | 1.000 | 1.000 |
| Females | pWW4 | 0.905 | 1.000 |

**References**

1. Ellis, V. A. *et al.* Explaining prevalence, diversity and host specificity in a community of avian haemosporidian parasites. *Oikos* **129**, 1314–1329 (2020).

2. Fletcher, K., Träff, J. & Gustafsson, L. Importance of infection of haemosporidia blood parasites during different life history stages for long-term reproductive fitness of collared flycatchers. *J. Avian Biol.* **50**, (2019).

3. Loiseau, C. *et al.* Host and habitat specialization of avian malaria in Africa. *Mol. Ecol.* **21**, 431–441 (2012).

4. Beadell, J. S. *et al.* Host associations and evolutionary relationships of avian blood parasites from West Africa. *Int. J. Parasitol.* **39**, 257–266 (2009).

5. Loiseau, C. *et al.* Insularity effects on the assemblage of the blood parasite community of the birds from the Gulf of Guinea. *J. Biogeogr.* **44**, 2607–2617 (2017).

6. Lutz, H. L. *et al.* Parasite prevalence corresponds to host life history in a diverse assemblage of Afrotropical birds and haemosporidian parasites. *PLoS ONE* **10**, (2015).

7. Emmenegger, T. *et al.* Population- and age-specific patterns of haemosporidian assemblages and infection levels in European bee-eaters (*Merops apiaster*). *Int. J. Parasitol.* **50**, 1125–1131 (2020).

8. Fourcade, Y., Keišs, O., Richardson, D. S. & Secondi, J. Continental-scale patterns of pathogen prevalence: A case study on the corncrake. *Evol. Appl.* **7**, 1043–1055 (2014).

9. Jones, W. *et al.* Interspecific transfer of parasites following a range-shift in *Ficedula* flycatchers. *Ecol. Evol.* **8**, 12183–12192 (2018).

10. Kulma, K., Low, M., Bensch, S. & Qvarnström, A. Malaria-infected female collared flycatchers (*Ficedula albicollis*) do not pay the cost of late breeding. *PLoS ONE* **9**, e85822 (2014).

11. Kulma, K., Low, M., Bensch, S. & Qvarnström, A. Malaria infections reinforce competitive asymmetry between two Ficedula flycatchers in a recent contact zone. *Mol. Ecol.* **22**, 4591–4601 (2013).

12. Radwan, J. *et al.* MHC diversity, malaria and lifetime reproductive success in collared flycatchers. *Mol. Ecol.* **21**, 2469–2479 (2012).

13. Szöllősi, E., Rosivall, B., Hasselquist, D. & Török, J. The effect of parental quality and malaria infection on nestling performance in the Collared Flycatcher (*Ficedula albicollis*). *J. Ornithol.* **150**, 519–527 (2009).

14. Hellgren, O. *et al.* Circannual variation in blood parasitism in a sub-Saharan migrant passerine bird, the garden warbler. *J. Evol. Biol.* **26**, 1047–1059 (2013).

15. Harvey, J. A. & Voelker, G. Avian haemosporidian detection across source materials: prevalence and genetic diversity. *Parasitol. Res.* **116**, 3361–3371 (2017).

16. Hellgren, O. *et al.* Detecting shifts of transmission areas in avian blood parasites - A phylogenetic approach. *Mol. Ecol.* **16**, 1281–1290 (2007).

17. Lauron, E. J. *et al.* Coevolutionary patterns and diversification of avian malaria parasites in African sunbirds (Family Nectariniidae). *Parasitology* **142**, 635–647 (2015).

18. Szöllősi, E. *et al.* *Haemoproteus* infection status of collared flycatcher males changes within a breeding season. *Parasitol. Res.* **115**, 4663–4672 (2016).

19. Ricklefs, R. E. & Fallon, S. M. Diversification and host switching in avian malaria parasites. *Proc. R. Soc. B Biol. Sci.* **269**, 885–892 (2002).

20. Beadell, J. S. *et al.* Prevalence and differential host-specificity of two avian blood parasite genera in the Australo-Papuan region. *Mol. Ecol.* **13**, 3829–3844 (2004).

21. Svensson, L. M. E., Ruegg, K. C., Sekercioglu, C. H. & Sehgal, R. N. M. Widespread and structured distributions of blood parasite haplotypes across a migratory divide of the Swainsons thrush (*Catharus ustulatus*). *J. Parasitol.* **93**, 1488–1495 (2007).

22. Oakgrove, K. S. *et al.* Distribution, diversity and drivers of blood-borne parasite co-infections in Alaskan bird populations. *Int. J. Parasitol.* **44**, 717–727 (2014).

23. DeBrock, S., Cohen, E., Balasubramanian, S., Marra, P. P. & Hamer, S. A. Characterization of the *Plasmodium* and *Haemoproteus* parasite community in temperate-tropical birds during spring migration. *Int. J. Parasitol. Parasites Wildl.* **15**, 12–21 (2021).

24. Gupta, P., Vishnudas, C. K., Ramakrishnan, U., Robin, V. V. & Dharmarajan, G. Geographical and host species barriers differentially affect generalist and specialist parasite community structure in a tropical sky-island archipelago. *Proc. R. Soc. B Biol. Sci.* **286**, (2019).

25. Silva-Iturriza, A., Ketmaier, V. & Tiedemann, R. Prevalence of avian haemosporidian parasites and their host fidelity in the central Philippine islands. *Parasitol. Int.* **61**, 650–657 (2012).

26. Ivanova, K., Zehtindjiev, P., Mariaux, J. & Georgiev, B. B. Genetic diversity of avian haemosporidians in Malaysia: Cytochrome b lineages of the genera *Plasmodium* and *Haemoproteus* (Haemosporida) from Selangor. *Infect. Genet. Evol.* **31**, 33–39 (2015).

27. Zehtindjiev, P., Ivanova, K., Mariaux, J. & Georgiev, B. B. First data on the genetic diversity of avian haemosporidians in China: Cytochrome b lineages of the genera *Plasmodium* and *Haemoproteus* (Haemosporida) from Gansu Province. *Parasitol. Res.* **112**, 3509–3515 (2013).

28. Garamszegi, L. Z. *et al.* Malaria parasites, immune challenge, MHC variability, and predator avoidance in a passerine bird. *Behav. Ecol.* **26**, 1292–1302 (2015).

29. Krizanauskiene, A. *et al.* Variation in host specificity between species of avian hemosporidian parasites: evidence from parasite morphology and cytochrome B gene sequences. *J. Parasitol.* **92**, 1319–24 (2006).

30. Peev, S. *et al.* Haemosporidian blood parasite diversity and prevalence in the semi-collared flycatcher (*Ficedula semitorquata*) from the eastern Balkans. *Parasitol. Int.* **65**, 613–617 (2016).

31. López, G., Muñoz, J., Soriguer, R. & Figuerola, J. Increased endoparasite infection in late-arriving individuals of a trans-Saharan passerine migrant bird. *PLoS ONE* **8**, e61236 (2013).

32. Mata, V. A., da Silva, L. P., Lopes, R. J. & Drovetski, S. V. The Strait of Gibraltar poses an effective barrier to host-specialised but not to host-generalised lineages of avian Haemosporidia. *Int. J. Parasitol.* **45**, 711–719 (2015).

33. Musa, S., Mackenstedt, U., Woog, F. & Dinkel, A. Avian malaria on Madagascar: prevalence, biodiversity and specialization of haemosporidian parasites. *Int. J. Parasitol.* **49**, 199–210 (2019).

34. Levin, I. I. *et al.* Multiple lineages of avian malaria parasites (*Plasmodium*) in the Galapagos Islands and evidence for arrival via migratory birds. *Conserv. Biol.* **27**, 1366–1377 (2013).

35. Durrant, K. L. *et al.* Avian hematozoa in South America: A comparison of temperate and tropical zones. *Ornithol. Monogr.* 98–111 (2006) doi:10.2307/40166831.

36. Fecchio, A. *et al.* Avian host composition, local speciation and dispersal drive the regional assembly of avian malaria parasites in South American birds. *Mol. Ecol.* **28**, 2681–2693 (2019).

37. Fecchio, A. *et al.* Higher infection probability of haemosporidian parasites in Blue-black Grassquits (*Volatinia jacarina*) inhabiting native vegetation across Brazil. *Parasitol. Int.* **80**, 102204 (2021).

38. Palinauskas, V. *et al.* Description of the first cryptic avian malaria parasite, *Plasmodium homocircumflexum* n. sp., with experimental data on its virulence and development in avian hosts and mosquitoes. *Int. J. Parasitol.* **45**, 51–62 (2015).

39. Piersma, T. & van der Velde, M. Dutch House Martins *Delichon urbicum* gain blood parasite infections over their lifetime, but do not seem to suffer. *J. Ornithol.* **153**, 907–912 (2012).

40. Valkiūnas, G. *et al.* *Plasmodium delichoni* n. sp.: description, molecular characterisation and remarks on the exoerythrocytic merogony, persistence, vectors and transmission. *Parasitol. Res.* **115**, 2625–2636 (2016).

41. Beadell, J. S. *et al.* Global phylogeographic limits of Hawaii’s avian malaria. *Proc. R. Soc. B Biol. Sci.* **273**, 2935–2944 (2006).

42. Schmid, S. *et al.* Avian malaria on Madagascar: bird hosts and putative vector mosquitoes of different *Plasmodium* lineages. *Parasit. Vectors* **10**, 1–7 (2017).

43. Ishtiaq, F., Beadell, J. S., Warren, B. H. & Fleischer, R. C. Diversity and distribution of avian haematozoan parasites in the western Indian Ocean region: A molecular survey. *Parasitology* **139**, 221–231 (2012).

44. Bensch, S. *et al.* Temporal dynamics and diversity of avian malaria parasites in a single host species. in *Journal of Animal Ecology* vol. 76 112–122 (John Wiley & Sons, Ltd, 2007).

45. Ivanova, K., Zehtindjiev, P., Mariaux, J., Dimitrov, D. & Georgiev, B. B. Avian haemosporidians from rain forests in Madagascar: Molecular and morphological data of the genera *Plasmodium*, *Haemoproteus* and *Leucocytozoon*. *Infect. Genet. Evol.* **58**, 115–124 (2018).

46. Chasar, A. *et al.* Prevalence and diversity patterns of avian blood parasites in degraded African rainforest habitats. *Mol. Ecol.* **18**, 4121–4133 (2009).

47. Bonneaud, C. *et al.* The prevalence of avian *Plasmodium* is higher in undisturbed tropical forests of Cameroon. *J. Trop. Ecol.* **25**, 439–447 (2009).

48. Loiseau, C. *et al.* Spatial variation of haemosporidian parasite infection in african rainforest bird species. *J. Parasitol.* **96**, 21–29 (2010).

49. Dimitrov, D., Zehtindjiev, P. & Bensch, S. Genetic diversity of avian blood parasites in SE Europe: Cytochrome b lineages of the genera *Plasmodium* and *Haemoproteus* (Haemosporida) from Bulgaria. *Acta Parasitol.* **55**, 201–209 (2010).

50. Garcia-Longoria, L., Marzal, A., De Lope, F. & Garamszegi, L. Host-parasite interaction explains variation in the prevalence of avian haemosporidians at the community level. *PLoS ONE* **14**, (2019).

51. Hahn, S. *et al.* Spatially different annual cycles but similar haemosporidian infections in distant populations of collared sand martins. *BMC Zool.* **6**, 1–11 (2021).

52. von Rönn, J. A. C., Harrod, C., Bensch, S. & Wolf, J. B. W. Transcontinental migratory connectivity predicts parasite prevalence in breeding populations of the European barn swallow. *J. Evol. Biol.* **28**, 535–546 (2015).

53. van Rooyen, J., Jenkins, T., Lahlah, N. & Christe, P. North-African house martins endure greater haemosporidian infection than their European counterparts. *J. Avian Biol.* **45**, 450–456 (2014).

54. Dimitrov, D., Ilieva, M., Ivanova, K., Brlík, V. & Zehtindjiev, P. Detecting local transmission of avian malaria and related haemosporidian parasites (Apicomlexa, Haemosporida) at a Special Protection Area of Natura 2000 network. *Parasitol. Res.* **117**, 2187–2199 (2018).

55. Ciloglu, A. *et al.* Prevalence and genetic diversity of avian haemosporidian parasites at an intersection point of bird migration routes: Sultan Marshes National Park, Turkey. *Acta Trop.* **210**, 105465 (2020).

56. Illera, J. C., Fernández-Álvarez, Á., Hernández-Flores, C. N. & Foronda, P. Unforeseen biogeographical patterns in a multiple parasite system in Macaronesia. *J. Biogeogr.* **42**, 1858–1870 (2015).

57. Drovetski, S. V. *et al.* Does the niche breadth or trade-off hypothesis explain the abundance-occupancy relationship in avian Haemosporidia? *Mol. Ecol.* **23**, 3322–3329 (2014).

58. Kim, K. S., Tsuda, Y. & Yamada, A. Bloodmeal identification and detection of avian malaria parasite from mosquitoes (Diptera: Culicidae) inhabiting coastal areas of Tokyo Bay, Japan. *J. Med. Entomol.* **46**, 1230–1234 (2009).

59. Marzal, A., Bensch, S., Reviriego, M., Balbontin, J. & De Lope, F. Effects of malaria double infection in birds: One plus one is not two. *J. Evol. Biol.* **21**, 979–987 (2008).

60. Mendes, L. *et al.* Hidden haemosporidian infections in Ruffs (Philomachus pugnax) staging in Northwest Europe en route from Africa to Arctic Europe. *Parasitol. Res.* **112**, 2037–2043 (2013).

61. Rojo, M. À., Campos, F., Santamaria, T. & Hernàndez, M. À. Haemosporidians in Iberian bluethroats *Luscinia svecica*. *Ardeola* **61**, 135–143 (2014).

62. Pérez-Rodríguez, A., de la Hera, I., Bensch, S. & Pérez-Tris, J. Evolution of seasonal transmission patterns in avian blood-borne parasites. *Int. J. Parasitol.* **45**, 605–611 (2015).

63. Svoboda, A. *et al.* Blood parasite prevalence in the Bluethroat is associated with subspecies and breeding habitat. *J. Ornithol.* **156**, 371–380 (2015).

64. Stanković, D., Jönsson, J. & Raković, M. Diversity of avian blood parasites in wild passerines in Serbia with special reference to two new lineages. *J. Ornithol.* **160**, 545–555 (2019).

65. Zehtindjiev, P. *et al.* Dynamics of parasitemia of malaria parasites in a naturally and experimentally infected migratory songbird, the great reed warbler Acrocephalus arundinaceus. *Exp. Parasitol.* **119**, 99–110 (2008).

66. Zehtindjiev, P. *et al.* Occurrence of haemosporidian parasites in the paddyfield warbler, *Acrocephalus agricola* (Passeriformes, Sylviidae). *Acta Parasitol.* **54**, 295–300 (2009).

67. Ventim, R. *et al.* Host-parasite associations and host-specificity in haemoparasites of reed bed passerines. *Parasitology* **139**, 310–316 (2012).

68. Bonneaud, C., Pérez-Tris, J., Federici, P., Chastel, O. & Sorci, G. Major histocompatibility alleles associated with local resistance to malaria in a passerine. *Evolution* **60**, 383 (2006).

69. Cosgrove, C. L., Wood, M. J., Day, K. P. & Sheldon, B. C. Seasonal variation in *Plasmodium* prevalence in a population of blue tits *Cyanistes caeruleus*. *J. Anim. Ecol.* **77**, 540–548 (2008).

70. Ayadi, T. *et al.* Diversity, prevalence and host specificity of avian parasites in southern Tunisian oases. *Parasitology* **145**, 971–978 (2018).

71. Dubiec, A. *et al.* Differential prevalence and diversity of haemosporidian parasites in two sympatric closely related non-migratory passerines. *Parasitology* **143**, 1320–1329 (2016).

72. Emmenegger, T. *et al.* Host migration strategy and blood parasite infections of three sparrow species sympatrically breeding in Southeast Europe. *Parasitol. Res.* **117**, 3733–3741 (2018).

73. Ferrer, E. S., García-Navas, V., Sanz, J. J. & Ortego, J. Molecular characterization of avian malaria parasites in three Mediterranean blue tit (*Cyanistes caeruleus*) populations. *Parasitol. Res.* **111**, 2137–2142 (2012).

74. Ferraguti, M. *et al.* Ecological determinants of avian malaria infections: An integrative analysis at landscape, mosquito and vertebrate community levels. *J. Anim. Ecol.* **87**, 727–740 (2018).

75. Knowles, S. C. L., Palinauskas, V. & Sheldon, B. C. Chronic malaria infections increase family inequalities and reduce parental fitness: Experimental evidence from a wild bird population. *J. Evol. Biol.* **23**, 557–569 (2010).

76. Knowles, S. C. L. *et al.* Molecular epidemiology of malaria prevalence and parasitaemia in a wild bird population. *Mol. Ecol.* **20**, 1062–1076 (2011).

77. Gutiérrez-López, R. *et al.* Do mosquitoes transmit the avian malaria-like parasite *Haemoproteus*? An experimental test of vector competence using mosquito saliva. *Parasit. Vectors* **9**, 1–7 (2016).

78. Kim, K. S. & Tsuda, Y. Seasonal changes in the feeding pattern of *Culex pipiens pallens* govern the transmission dynamics of multiple lineages of avian malaria parasites in Japanese wild bird community. *Mol. Ecol.* **19**, 5545–5554 (2010).

79. Lynton-Jenkins, J. G. *et al.* Contrasting the seasonal and elevational prevalence of generalist avian haemosporidia in co-occurring host species. *Ecol. Evol.* **10**, 6097–6111 (2020).

80. Loiseau, C. *et al.* Antagonistic effects of a MHC class I allele on malaria-infected house sparrows. *Ecol. Lett.* **11**, 258–265 (2008).

81. Marzal, A. *et al.* Diversity, loss, and gain of malaria parasites in a globally invasive bird. *PLoS ONE* **6**, e21905 (2011).

82. Martinsen, E. S., Paperna, I. & Schall, J. J. Morphological versus molecular identification of avian Haemosporidia: An exploration of three species concepts. *Parasitology* **133**, 279–288 (2006).

83. Martínez De La Puente, J. *et al.* Avian malaria parasites in the last supper: Identifying encounters between parasites and the invasive Asian mosquito tiger and native mosquito species in Italy. *Malar. J.* **14**, 1–7 (2015).

84. Neto, J. M. *et al.* Seasonal dynamics of haemosporidian (Apicomplexa, Haemosporida) parasites in house sparrows *Passer domesticus* at four European sites: comparison between lineages and the importance of screening methods. *Int. J. Parasitol.* **50**, 523–532 (2020).

85. Podmokła, E. *et al.* Avian malaria is associated with increased reproductive investment in the blue tit. *J. Avian Biol.* **45**, 219–224 (2014).

86. Podmokła, E. *et al.* Determinants of prevalence and intensity of infection with malaria parasites in the Blue Tit. *J. Ornithol.* **155**, 721–727 (2014).

87. Szöllősi, E. *et al.* Determinants of distribution and prevalence of avian malaria in blue tit populations across Europe: Separating host and parasite effects. *J. Evol. Biol.* **24**, 2014–2024 (2011).

88. Schumm, Y. R. *et al.* Blood parasites in Passeriformes in central Germany: Prevalence and lineage diversity of Haemosporida (*Haemoproteus*, *Plasmodium* and *Leucocytozoon*) in six common songbirds. *PeerJ* **2019**, e6259 (2019).

89. Shurulinkov, P., Spasov, L., Stoyanov, G. & Chakarov, N. Blood parasite infections in a wild population of ravens (*Corvus corax*) in Bulgaria. *Malar. J.* **17**, 33 (2018).

90. Wood, M. J. *et al.* Within-population variation in prevalence and lineage distribution of avian malaria in blue tits, *Cyanistes caeruleus*. *Mol. Ecol.* **16**, 3263–3273 (2007).

91. van Rooyen, J., Lalubin, F., Glaizot, O. & Christe, P. Altitudinal variation in haemosporidian parasite distribution in great tit populations. *Parasit. Vectors* **6**, 1–10 (2013).

92. van Rooyen, J., Lalubin, F., Glaizot, O. & Christe, P. Avian haemosporidian persistence and co-infection in great tits at the individual level. *Malar. J.* **12**, 1–8 (2013).

93. Zehtindjiev, P. *et al.* Haemosporidian infections in skylarks (*Alauda arvensis*): A comparative PCR-based and microscopy study on the parasite diversity and prevalence in southern Italy and the Netherlands. *Eur. J. Wildl. Res.* **58**, 335–344 (2012).

94. Santiago-Alarcon, D., MacGregor-Fors, I., Kühnert, K., Segelbacher, G. & Schaefer, H. M. Avian haemosporidian parasites in an urban forest and their relationship to bird size and abundance. *Urban Ecosyst.* **19**, 331–346 (2016).

95. López-Calderón, C. *et al.* Rainfall at African wintering grounds predicts age-specific probability of haemosporidian infection in a migratory passerine bird. *Ibis* **161**, 759–769 (2019).

96. Martinsen, E. S., Perkins, S. L. & Schall, J. J. A three-genome phylogeny of malaria parasites (*Plasmodium* and closely related genera): Evolution of life-history traits and host switches. *Mol. Phylogenet. Evol.* **47**, 261–273 (2008).

97. Hellgren, O., Križanauskiene, A., Valkiūnas, G. & Bensch, S. Diversity and phylogeny of mitochondrial cytochrome B lineages from six morphospecies of avian *Haemoproteus* (Haemosporida: Haemoproteidae). *J. Parasitol.* **93**, 889–896 (2007).

98. Palinauskas, V. *et al.* Molecular characterization and distribution of *Haemoproteus minutus* (Haemosporida, Haemoproteidae): A pathogenic avian parasite. *Parasitol. Int.* **62**, 358–363 (2013).

99. Valkiūnas, G. *et al.* Further observations on in vitro hybridization of hemosporidian parasites: Patterns of ookinete development in *Haemoproteus* spp. *J. Parasitol.* **99**, 124–136 (2013).

100. Waldenström, J., Bensch, S., Kiboi, S., Hasselquist, D. & Ottosson, U. Cross-species infection of blood parasites between resident and migratory songbirds in Africa. *Mol. Ecol.* **11**, 1545–1554 (2002).

101. Nilsson, E. *et al.* Multiple cryptic species of sympatric generalists within the avian blood parasite *Haemoproteus majoris*. *J. Evol. Biol.* **29**, 1812–1826 (2016).

102. Scordato, E. S. C. & Kardish, M. R. Prevalence and beta diversity in avian malaria communities: Host species is a better predictor than geography. *J. Anim. Ecol.* **83**, 1387–1397 (2014).

103. Imura, T. *et al.* Prevalence of avian haematozoa in wild birds in a high-altitude forest in Japan. *Vet. Parasitol.* **183**, 244–248 (2012).

104. Durrant, K. L. *et al.* Variation in haematozoan parasitism at local and landscape levels in the red-billed quelea *Quelea quelea*. *J. Avian Biol.* **38**, 662–671 (2007).

105. Biedrzycka, A. *et al.* Blood parasites shape extreme major histocompatibility complex diversity in a migratory passerine. *Mol. Ecol.* **27**, 2594–2603 (2018).

106. Ortego, J., Cordero, P. J., Aparicio, J. M. & Calabuig, G. Consequences of chronic infections with three different avian malaria lineages on reproductive performance of Lesser Kestrels (*Falco naumanni*). *J. Ornithol.* **149**, 337–343 (2008).

107. Ortego, J., Calabuig, G., Cordero, P. J. & Aparicio, J. M. Genetic characterization of avian malaria (Protozoa) in the endangered lesser kestrel, *Falco naumanni*. *Parasitol. Res.* **101**, 1153–1156 (2007).

108. Pérez-Rodríguez, A., de la Puente, J., Onrubia, A. & Pérez-Tris, J. Molecular characterization of haemosporidian parasites from kites of the genus *Milvus* (Aves: Accipitridae). *Int. J. Parasitol.* **43**, 381–387 (2013).

109. Schumm, Y. R. *et al.* Prevalence and genetic diversity of avian haemosporidian parasites in wild bird species of the order Columbiformes. *Parasitol. Res.* **120**, 1405–1420 (2021).

110. Wiersch, S. C., Lubjuhn, T., Maier, W. A. & Kampen, H. Haemosporidian infection in passerine birds from Lower Saxony. *J. Ornithol.* **148**, 17–24 (2007).

111. Okanga, S., Cumming, G. S., Hockey, P. A. R., Nupen, L. & Peters, J. L. Host specificity and co-speciation in avian haemosporidia in the Western Cape, South Africa. *PLoS ONE* **9**, e86382 (2014).

112. Ewen, J. G. *et al.* Establishment of exotic parasites: The origins and characteristics of an avian malaria community in an isolated island avifauna. *Ecol. Lett.* **15**, 1112–1119 (2012).

113. Schoener, E. R., Tompkins, D. M., Parker, K. A., Howe, L. & Castro, I. Presence and diversity of mixed avian *Plasmodium* spp. infections in introduced birds whose distribution overlapped with threatened New Zealand endemic birds. *N. Z. Vet. J.* **68**, 101–106 (2020).

114. Turcotte, A., Bélisle, M., Pelletier, F. & Garant, D. Environmental determinants of haemosporidian parasite prevalence in a declining population of Tree swallows. *Parasitology* **145**, 961–970 (2018).

115. Theodosopoulos, A. N., Grabenstein, K. C., Bensch, S. & Taylor, S. A. A highly invasive malaria parasite has expanded its range to non-migratory birds in North America. *Biol. Lett.* **17**, (2021).

116. Quillfeldt, P. *et al.* Hemosporidian blood parasites in seabirds - A comparative genetic study of species from Antarctic to tropical habitats. *Naturwissenschaften* **97**, 809–817 (2010).

117. Marzal, A., García-Longoria, L., Cárdenas Callirgos, J. M. & Sehgal, R. N. M. Invasive avian malaria as an emerging parasitic disease in native birds of Peru. *Biol. Invasions* **17**, 39–45 (2015).

118. Dimitrov, D., Valkiūnas, G., Zehtindjiev, P., Ilieva, M. & Bensch, S. Molecular characterization of haemosporidian parasites (Haemosporida) in yellow wagtail (*Motacilla flava*), with description of in vitro ookinetes of *Haemoproteus motacillae*. *Zootaxa* **3666**, 369–381 (2013).

119. Ferraguti, M., Martínez-De La Puente, J., Ruiz, S., Soriguer, R. & Figuerola, J. On the study of the transmission networks of blood parasites from SW Spain: Diversity of avian haemosporidians in the biting midge *Culicoides circumscriptus* and wild birds. *Parasit. Vectors* **6**, 1–7 (2013).

120. Hellgren, O. The occurrence of haemosporidian parasites in the Fennoscandian bluethroat (*Luscinia svecica*) population. *J. Ornithol.* **146**, 55–60 (2005).

121. Pardal, S. *et al.* Shorebird low spillover risk of mosquito-borne pathogens on Iberian wetlands. *J. Ornithol.* **155**, 549–554 (2014).

122. Santiago-Alarcon, D., Bloch, R., Rolshausen, G., Schaefer, H. M. & Segelbacher, G. Prevalence, diversity, and interaction patterns of avian haemosporidians in a four-year study of blackcaps in a migratory divide. *Parasitology* **138**, 824–835 (2011).

123. Synek, P. *et al.* Haemosporidian parasites of a European passerine wintering in South Asia: Diversity, mixed infections and effect on host condition. *Parasitol. Res.* **112**, 1667–1677 (2013).

124. Palinauskas, V., Valkiūnas, G., Bolshakov, C. V. & Bensch, S. *Plasmodium relictum* (lineage SGS1) and <i<Plasmodium ashfordi</i> (lineage GRW2): The effects of the co-infection on experimentally infected passerine birds. *Exp. Parasitol.* **127**, 527–533 (2011).

125. Glaizot, O. *et al.* High prevalence and lineage diversity of avian malaria in wild populations of great tits (Parus major) and mosquitoes (Culex pipiens). *PloS One* **7**, e34964 (2012).

126. Huang, X. *et al.* A new protocol for absolute quantification of haemosporidian parasites in raptors and comparison with current assays. *Parasit. Vectors* **13**, 1–9 (2020).

127. Huang, X., Dong, L., Zhang, C. & Zhang, Y. Genetic diversity, temporal dynamics, and host specificity in blood parasites of passerines in north China. *Parasitol. Res.* **114**, 4513–4520 (2015).

128. Isaksson, C., Sepil, I., Baramidze, V. & Sheldon, B. C. Explaining variance of avian malaria infection in the wild: The importance of host density, habitat, individual life-history and oxidative stress. *BMC Ecol.* **13**, 1–11 (2013).

129. Kim, K. S., Tsuda, Y., Sasaki, T., Kobayashi, M. & Hirota, Y. Mosquito blood-meal analysis for avian malaria study in wild bird communities: Laboratory verification and application to *Culex sasai* (Diptera: Culicidae) collected in Tokyo, Japan. *Parasitol. Res.* **105**, 1351–1357 (2009).

130. Liu, B., Deng, Z., Huang, W., Dong, L. & Zhang, Y. High prevalence and narrow host range of haemosporidian parasites in Godlewski’s bunting (*Emberiza godlewskii*) in northern China. *Parasitol. Int.* **69**, 121–125 (2019).

131. Loiseau, C. *et al.* Plasmodium relictum infection and MHC diversity in the house sparrow (*Passer domesticus*). *Proc. R. Soc. B Biol. Sci.* **278**, 1264–1272 (2011).

132. Martínez, J. *et al.* A restriction site to differentiate *Plasmodium* and *Haemoproteus* infections in birds: On the inefficiency of general primers for detection of mixed infections. *Parasitology* **136**, 713–722 (2009).

133. Muriel, J. *et al.* Molecular characterization of avian malaria in the spotless starling (*Sturnus unicolor*). *Parasitol. Res.* **117**, 919–928 (2018).

134. Palinauskas, V., Kosarev, V., Shapoval, A., Bensch, S. & Valkiūnas, G. Comparison of mitochondrial cytochrome b lineages and morphospecies of two avian malaria parasites of the subgenera *Haemamoeba* and </i>Giovannolaia</i> (Haemosporida: Plasmodiidae). *Zootaxa* **1626**, 39–50 (2007).

135. Scaglione, F. E. *et al.* Prevalence of new and known species of haemoparasites in feral pigeons in northwest Italy. *Malar. J.* **14**, 1–5 (2015).

136. Schmid, S., Fachet, K., Dinkel, A., Mackenstedt, U. & Woog, F. Carrion crows (*Corvus corone*) of southwest Germany: Important hosts for haemosporidian parasites. *Malar. J.* **16**, 1–12 (2017).

137. Seimon, T. A. *et al.* Avian hemosporidian parasite lineages in four species of freeranging migratory waterbirds from Mongolia, 2008. *J. Wildl. Dis.* **52**, 682–687 (2016).

138. Ventim, R. *et al.* Characterization of haemosporidian infections in warblers and sparrows at south-western European reed beds. *J. Ornithol.* **153**, 505–512 (2012).

139. Stjernman, M., Råberg, L. & Nilsson, J. Å. Maximum host survival at intermediate parasite infection intensities. *PLoS ONE* **3**, e2463 (2008).

140. Zagalska-Neubauer, M. & Bensch, S. High prevalence of *Leucocytozoon* parasites in fresh water breeding gulls. *J. Ornithol.* **157**, 525–532 (2016).

141. Chaisi, M. E., Osinubi, S. T., Dalton, D. L. & Suleman, E. Occurrence and diversity of avian haemosporidia in Afrotropical landbirds. *Int. J. Parasitol. Parasites Wildl.* **8**, 36–44 (2019).

142. Ciloglu, A. *et al.* Investigation of avian haemosporidian parasites from raptor birds in Turkey, with molecular characterisation and microscopic confirmation. *Folia Parasitol. (Praha)* **63**, 23 (2016).

143. Harl, J. *et al.* Geographic and host distribution of haemosporidian parasite lineages from birds of the family Turdidae. *Malar. J.* **19**, 1–35 (2020).

144. Krone, O. *et al.* Haemosporidian blood parasites in European birds of prey and owls. *J. Parasitol.* **94**, 709–715 (2008).

145. Kubacka, J., Gerlée, A., Foucher, J., Korb, J. & Podmokła, E. Correlates of blood parasitism in a threatened marshland passerine: Infection by kinetoplastids of the genus *Trypanosoma* is related to landscape metrics of habitat edge. *Parasitology* **146**, 1036–1046 (2019).

146. Hanel, J. *et al.* Blood parasites in northern goshawk (*Accipiter gentilis*) with an emphasis to *Leucocytozoon toddi*. *Parasitol. Res.* **115**, 263–270 (2016).

147. Synek, P. *et al.* Haemosporidian infections in the Tengmalm’s Owl (*Aegolius funereus*) and potential insect vectors of their transmission. *Parasitol. Res.* **115**, 291–298 (2016).

148. Bensch, S., Jönsson, J. & Copete, J. L. Low prevalence of *Haemoproteus* infections in chiffchaffs. *Parasitology* **139**, 302–309 (2012).

149. Bensch, S. & Akesson, S. Temporal and spatial variation of hematozoans in Scandinavian willow warblers. *J. Parasitol.* **89**, 388–391 (2003).

150. Dimitrov, D. *et al.* Molecular characterisation of three avian haemoproteids (Haemosporida, Haemoproteidae), with the description of *Haemoproteus* (*Parahaemoproteus*) *palloris* n. sp. *Syst. Parasitol.* **93**, 431–449 (2016).
